# Cancer prevalence, incidence and mortality in people who experience incarceration in Ontario, Canada: A population-based retrospective cohort study

**DOI:** 10.1371/journal.pone.0171131

**Published:** 2017-02-22

**Authors:** Fiona G. Kouyoumdjian, Lucie Pivnick, Kathryn E. McIsaac, Andrew S. Wilton, Aisha Lofters, Stephen W. Hwang

**Affiliations:** 1 Centre for Urban Health Solutions, St. Michael’s Hospital, Toronto, Ontario, Canada; 2 Department of Family Medicine, McMaster University, Hamilton, Ontario, Canada; 3 Faculty of Medicine, McMaster University, Hamilton, Ontario, Canada; 4 Research Services, Nova Scotia Health Authority, Halifax, Nova Scotia, Canada; 5 Institute for Clinical Evaluative Sciences, Toronto, Ontario, Canada; 6 Department of Family and Community Medicine, St. Michael’s Hospital, Toronto, Ontario, Canada; Leibniz Institute for Prevention Research and Epidemiology BIPS, GERMANY

## Abstract

**Introduction:**

Evidence suggests that many risk factors for cancer are overrepresented in people who experience incarceration, and data on cancer epidemiology are limited for this population. We aimed to describe cancer prevalence, incidence and mortality in adults admitted to provincial custody in Ontario, Canada in 2000.

**Methods:**

We linked data on 48,166 adults admitted to provincial custody in Ontario in 2000 with Ontario Cancer Registry data to 2012. We calculated cancer prevalence in the 10 years prior to admission to custody in 2000, incidence between 2000 and 2012 and mortality between 2000 and 2011. Standardized for age, we calculated incidence and mortality ratios by sex compared to the general population of Ontario.

**Results:**

The 10-year cancer prevalence was 0.4% in men and 0.6% in women at admission to provincial custody in 2000. Between 2000 and 2012, 2.6% of men and 2.8% of women were diagnosed with new cancer. The standardized incidence ratio for cancer was 1.0 (95% CI 0.9–1.0) for men and 0.9 (95% CI 0.7–1.0) for women compared to the general population, and was significantly increased for cervical, head and neck, liver and lung cancers. The standardized mortality ratio was 1.6 (95% CI 1.4–1.7) in men and 1.4 (95% CI 1.0–1.9) in women, and was significantly increased for head and neck, liver, and lung cancers.

**Conclusions:**

There is an excess burden of cancer in people who experience incarceration. Cancer prevention should include people who experience incarceration, and the period of incarceration may offer an opportunity for intervention.

## Introduction

An estimated 11 million people are imprisoned worldwide at any given time [1]. In Canada, there are over 250,000 adult admissions each year to correctional facilities and about 40,000 people in correctional facilities each day [2–4].

People who experience incarceration in Canada and internationally have worse health than the general population across a range of indicators [5, 6]. The epidemiology of cancer in this population has not been well characterized, despite evidence indicating the overrepresentation of risk factors in prisoners for cancer incidence and progression [5, 6]. Specific data on prisoners in Canada reveal a high prevalence of current smoking [7, 8], alcohol use [9–11], and infections such as hepatitis B virus (HBV) [12–14], hepatitis C virus (HCV) [15, 16] and human immunodeficiency virus (HIV) [15, 17].

Data on cancer prevalence in prisoners may elucidate health care needs in custody and inform health care service planning in custody and at release. The self-reported lifetime prevalence of any cancer was 5% in men and 15% in women prisoners in New South Wales, Australia in 2001 [18] and 1.1% in men and 8.3% in women in local jails in the USA in 2002 [19]. The prevalence of current cancer was 1.3% in White men and 0.4% in Black men in the US 2004 Survey of Inmates in State Correctional Facilities [20], and 0.8% in men and 2.7% in women in maximum security facilities in New York State between 2009 and 2011 [21]. Based on US national survey data from 2002 to 2004, there was no significant difference in the self-reported prevalence of any cancer in people in jails or prisons compared to non-institutionalized adults after adjusting for age and sex [22]. Looking at specific cancers, however, this analysis identified that cervical cancer was significantly more common in women in jails or prisons compared to non-institutionalized women [22], which is consistent with the high prevalence of cervical cancer found in other studies of incarcerated women [23–29]. One study found a high prevalence of oral cancer in incarcerated men in Maharashtra, India [30].

Less is known about cancer incidence in people who experience incarceration, and most studies have been limited by a lack of data on persons at risk and person-time at risk [31–33]. One exception is a study of persons incarcerated in prisons in the state of Georgia in 1991 who were still alive in 1998, which found incidence rates per 1,000 person years of 3.1 for any cancer, 0.01 for cervical cancer, 0.1 for liver cancer, 0.8 for lung and bronchus cancer, 0.6 for prostate cancer, 0.32 for colorectal cancer, and 0.1 for kidney cancer [34]. Adjusted for age, race, sex, and year of diagnosis and compared to the general population, the standardized incidence ratio (SIR) for any cancer was significantly increased at 2.3 [34].

Regarding cancer mortality, several large US cohort studies have identified an increased standardized mortality ratio (SMR) due to cancer in people who experience incarceration compared to the general population after controlling for age, sex, and race, with SMRs of 1.2 to 1.9 [35–37]. This finding is consistent with results from a study by our research group in Ontario, Canada that found an SMR for cancer of 1.6 after controlling for age [38]. Other US data suggest that race may modify the effect of a history of incarceration on cancer mortality, with significantly increased SMRs for White men and significantly decreased SMRs for Black men incarcerated in North Carolina, which the authors suggest could be due to poor healthcare access in non-incarcerated Black men [39, 40]. In the study of persons in prison in Georgia in 1991 who were still alive in 1998, the SMR for any cancer was not increased, at 1.0 [34]. SMRs tend to be increased for liver cancer [39–41] and lung or bronchial cancer [39, 40], with differences in SMRs potentially attributable to the aforementioned effect modification by race [39, 40].

In summary, comparing prisoners with the general population, the prevalence of any cancer is similar and the prevalence of cervical cancer is high, the SMR for any cancer varies with evidence of effect modification by race and the SMR for some specific cancer sites tends to be high. Incidence data are limited and suggest that incidence of any cancer is high compared with the general population. However, the use of self-reported data for determining prevalence may limit internal validity [42], the lack of data on the full population of people who experience incarceration [34] and of appropriate denominators for incidence rates [31–33] limits the generalizability of incidence data, and the lack of incidence and mortality data for specific types of cancer precludes the implementation of measures to improve cancer morbidity and mortality in this population.

We aimed to describe cancer prevalence, incidence and mortality in people admitted to provincial custody in Ontario, Canada in 2000, and to compare cancer incidence and mortality between those admitted to provincial custody in 2000 and the general population.

## Methods

### Study cohort

We defined cohort members as all men and women admitted to provincial correctional facilities for adults in Ontario in 2000, whether remanded, *i*.*e*. admitted to custody but not yet sentenced, or incarcerated, *i*.*e*. sentenced; we use the term incarcerated in this paper to include both groups. We followed this cohort for incident cancer until December 31, 2012 and for death due to cancer until December 31, 2011 based on data that were available at the time of analysis. We were not able to identify people who moved out of Ontario during the period under study. During the period under study, there was no routine cancer screening program in place in provincial correctional facilities, but there was a routine clinical assessment by nursing staff at admission and by a physician within weeks of admission.

The Ontario Ministry of Community Safety and Correctional Services (MCSCS) provided demographic data, health card numbers, information on death while under supervision, and self-reported race. On each admission, correctional staff verified or requested a health card number from the Ontario Ministry of Health and Long-Term Care, for the purposes of physician billings and other health care use. Persons who were eligible for health care coverage (*i*.*e*. residents of the province) who did not have a valid health card number were provided with a temporary number while in custody.

### Data transfer and linkage

The MCSCS transferred data on 49,470 persons admitted to custody in 2000 to the Institute for Clinical Evaluative Sciences, an independent, nonprofit organization funded by the Ontario Ministry of Health and Long-Term Care. We linked eligible persons in the MCSCS data set to people in the Registered Persons Database, which is a roster of all people eligible for the Ontario Health Insurance Plan. For persons with a health card number provided by the MCSCS (N = 40,593), we used deterministic linkage by health card number. For persons with no health card number provided by the MCSCS, we used a validated algorithm for probabilistic linkage using name (or names for persons with multiple names or aliases), sex and date of birth, and staff conducted clerical review of matches as needed [43]. As explained in detail elsewhere [38], we excluded matches that were likely inaccurate (n = 208), for example when the sex was different in the MCSCS data and Registered Persons Database, or if there were data indicating health care use or incarceration after the identified date of death.

Through the Registered Persons Database, we accessed a unique encrypted health card number (IKN), which was used to identify individuals across health care databases. We used the IKN to access data on cancer from the Ontario Cancer Registry, which contains data on newly diagnosed cases of cancer (except non-melanoma skin cancer) and deaths due to cancer for residents of Ontario. Cancers were classified according to the International Classification of Diseases for Oncology, 3^rd^ edition, ICD-O-3 [44]. We obtained data on specific types of cancer based on hypotheses regarding increased risk in the incarcerated cohort and the most common cancers in the Canadian population [45] and using the following ICD-O-3 codes: breast (C50), cervical (C53), colorectal (C18-20), head and neck (C00-C14), liver (C22), lung (C34) and prostate (C61).

### General population comparator data

We accessed publicly available data from Statistics Canada [46] on incidence and mortality in 2006, as the midpoint of the follow up period. We accessed data on incident cases for Ontario (Table 103–0550) and data on deaths for Canada (Table 102–0522) since age stratum-specific data on mortality due to cancer were not available for Ontario, and data on population sizes for Ontario and Canada (Table 051–0001) [46].

### Analysis

For any cancer and specific cancers, we calculated the cancer prevalence as the number of cases in persons in the ten, five, and two years preceding initial admission to custody in 2000 divided by the total number of persons admitted to custody. For the cancer incidence rate, we divided the number of cases of new primary cancers diagnosed between the date of admission to custody in 2000 and December 31, 2012, by the person years at risk of incident cancer, which we defined as the difference between the date of admission to custody in 2000 and December 31, 2012, death, or the diagnosis of primary cancer (whichever occurred first), and we excluded persons with the same type of cancer in the ten years before admission to custody (N = 1). For the cancer mortality rate, we divided the number of deaths due to cancer between the date of admission to custody in 2000 and December 31, 2011 by the person years at risk of death, defined as the difference between the date of admission to custody in 2000 and December 31, 2011 or death (whichever occurred first). The risk period for death was censored at the end of 2011 because that was the most recent date for which cause of death was available in the Ontario Cancer Registry at the time of the analysis.

We used indirect standardization to adjust for age in calculating standardized incidence and mortality ratios compared to the general population [47], and we calculated confidence intervals assuming a Poisson distribution.

Analyses were performed using SAS version 9.4 and Stata version 12.

The study was approved by the Ministry of Community Safety and Correctional Services Research Committee and by the St. Michael’s Hospital Research Ethics Board. Consistent with Article 5.5A of the Canadian Tri-Council Policy regarding the secondary use of data [48], no written or verbal consent was obtained from participants for the secondary use of these data and this was approved by the St. Michael’s Hospital Research Ethics Board.

## Results

Of the 49,470 persons admitted to adult provincial correctional facilities in Ontario in 2000, we linked 48,166 persons (97.4%) with health administrative data, as explained in detail elsewhere [38]. Characteristics of the study sample are shown in Table 1.

**Table 1:** Characteristics of persons admitted to provincial custody in Ontario in 2000, N = 48,166, by sex.

		Men, N = 43,419		Women, N = 4,747	
		n	%	n	%
Age at baseline	15–19	4,054	9.3	411	8.7
	20–29	14,606	33.6	1,436	30.3
	30–39	13,878	32.0	1,779	37.5
	40–49	7,814	18.0	873	18.4
	50–59	2,336	5.4	191	4.0
	60+	731	1.7	58	1.2
Self-reported race at baseline	Aboriginal	3,005	6.9	460	9.7
	Black	5,374	12.4	596	12.6
	East Asian	545	1.3	35	0.7
	Hispanic	392	0.9	28	0.6
	South Asian	897	2.1	54	1.1
	South East Asian	725	1.7	70	1.5
	West Asian/Arab	572	1.3	26	0.5
	White	29,977	69.0	3,279	69.1
	Other*	1932	4.4	199	4.2
Neighbourhood income quintile at baseline	missing	7,862	18.1	910	19.2
	1 (lowest)	13,336	30.7	1,693	35.7
	2	8,381	19.3	858	18.1
	3	5,892	13.6	573	12.1
	4	4,674	10.8	393	8.3
	5 (highest)	3,274	7.5	320	6.7
Admissions to provincial custody 2000–2012	1	14,238	32.8	1,885	39.7
	2–4	14,678	33.8	1,392	29.3
	5+	14,503	33.4	1,470	31.0
Transferred to federal facility 2000–2012	no	38,680	89.1	4,480	94.4
	yes	4,739	10.9	267	5.6
Length of index incarceration in 2000	<1 month	28,062	64.6	3,792	79.9
	1 month- <3 months	8,133	18.7	596	12.6
	3 months- <6 months	4,439	10.2	239	5.0
	6 months- <1 year	1,981	4.6	95	2.0
	≥1 year	804	1.9	25	0.5
Total time in provincial custody 2000–2012	<1 month	13,682	31.5	2,221	46.8
	1 month- <3 months	6,971	16.1	787	16.6
	3 months- <6 months	6,072	14.0	628	13.2
	6 months- <1 year	6,132	14.1	534	11.2
	≥1 year	10,562	24.3	577	12.1

*Includes other self-reported race, unknown, or refused.

At the time of admission to custody in 2000, the 10-year cancer prevalence was 0.4% in men and 0.6% in women, with specific cancer sites shown in Table 2. Of the 190 people who were admitted to custody with prevalent cancer, 36.8% had been diagnosed with cancer within two years of admission, 32.6% between two and five years of admission, and 30.5% between five and 10 years of admission.

**Table 2:** Cancer prevalence in persons admitted to provincial custody in Ontario in 2000, by sex and primary cancer site.

Cancer site	Men N = 43,419, n (%)			Women N = 4,747, n (%)		
	10-year	5-year	2-year	10-year	5-year	2-year
All	160 (0.4)	110 (0.3)	61 (0.1)	30 (0.6)	22 (0.5)	9 (0.2)
Breast	-	-	-	7 (0.1)	6 (0.1)	≤5* (≤0.1)
Cervical	-	-	-	8 (0.2)	0 (0.0)	0 (0.0)
Colorectal	13 (0.0)	9 (0.0)	5 (0.0)	≤5* (≤0.1)	≤5* (≤0.1)	≤5* (≤0.1)
Head and neck	12 (0.0)	7 (0.0)	3 (0.0)	0 (0.0)	0 (0.0)	0 (0.0)
Liver	≤5* (0.0)	≤5* (0.0)	≤5* (0.0)	0 (0.0)	0 (0.0)	0 (0.0)
Lung	8 (0.0)	≤5* (0.0)	≤5* (0.0)	0 (0.0)	0 (0.0)	0 (0.0)
Prostate	24 (0.1)	19 (0.0)	10 (0.0)	-	-	-
Other	104 (0.2)	70 (0.2)	32 (0.1)	14 (0.3)	10 (0.2)	≤5* (≤0.1)

*To decrease the risk of identifying individuals, we indicated ≤5 as the number of people in cells in which there were 5 or fewer persons. For the percentage, we indicated the true percentage if the value didn’t change for numerators between 1 and 5, or else we used 5 as the numerator and indicated the value as less than or equal to the result.

Between 2000 and 2012, 2.6% of men and 2.8% of women were diagnosed with new primary cancers. As per Table 3, the most common types of incident cancer for men were lung, prostate, colorectal, and head and neck, while the most common types of cancer for women were breast, lung, and cervical.

**Table 3:** Cancer incidence and mortality in persons admitted to provincial custody in Ontario in 2000,* by sex and primary cancer site.

Cancer site	Incidence 2000 to 2012, n (cases per 1,000 person years)		Deaths 2000 to 2011, n (deaths per 1,000 person years)	
	Men	Women	Men	Women
All	1141 (2.2)	135 (2.4)	491 (1.0)	42 (0.8)
Breast	-	30 (0.5)	-	6 (0.1)
Cervical	-	18 (0.3)	-	≤5^†^ (≤0.1)
Colorectal	108 (0.2)	9 (0.2)	35 (0.1)	0 (0)
Head and neck	92 (0.2)	≤5^†^ (≤0.1)	26 (0.1)	≤5^†^ (≤0.1)
Liver	63 (0.1)	≤5^†^ (≤0.1)	44 (0.1)	≤5^†^ (≤0.1)
Lung	246 (0.5)	26 (0.5)	157 (0.3)	15 (0.3)
Prostate	162 (0.3)	-	9 (0.0)	-
Other	547 (0.9)	52 (0.9)	210 (0.4)	11 (0.2)

*Total population is 43,419 men and 4,747 women.

^†^To decrease the risk of identifying individuals in cells in which there were 5 or fewer persons, we indicated ≤5 as the number of people and we used 5 as the numerator to calculate the percentage and indicated the value as less than or equal to the result.

**Fig 1:**
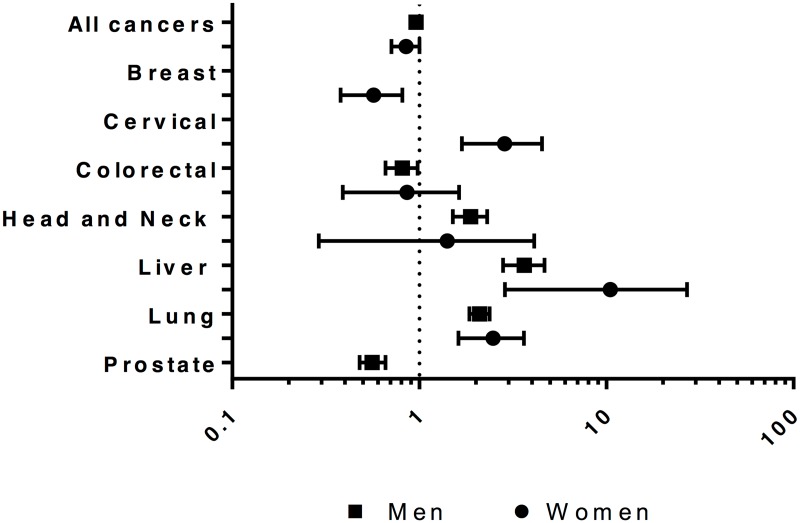
Standardized* incidence ratios for cancer for persons admitted to provincial custody in Ontario in 2000 compared to general population of Ontario in 2006, by sex and primary cancer site. *Standardized for age.

The SIR for any cancer was 1.0 (95% CI 0.9–1.0) for men and 0.9 (95% CI 0.7–1.0) for women in the cohort compared to the general population. The SIR was higher in men for lung, liver, and head and neck cancers, and lower for prostate and colorectal cancers (Fig 1). For women, the SIR was higher for lung, cervical, and liver cancers, and lower for breast cancer.

For cancers for which the SIR was significantly increased for cohort members compared to the general population, the relative risk of cancer was increased across most age strata, though there were no cases in several age strata for specific types of cancer (S1 Appendix).

**Fig 2:**
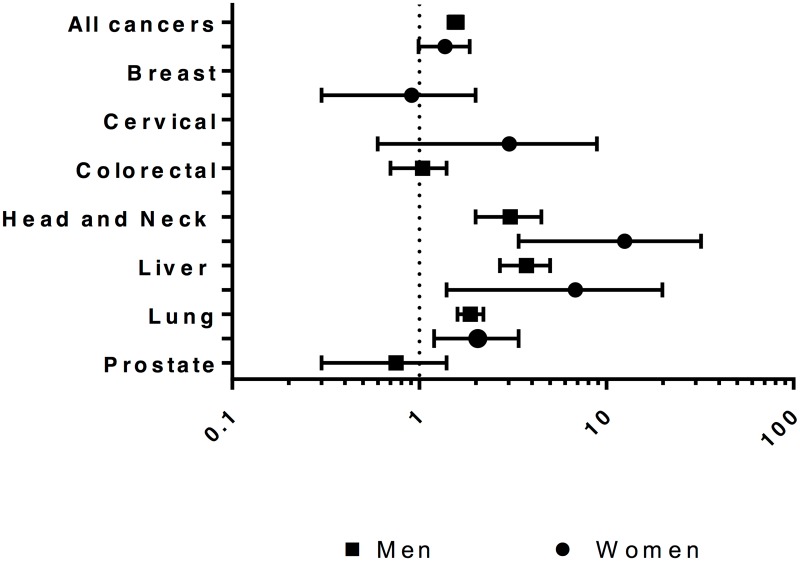
Standardized* mortality ratios† for cancer for persons admitted to provincial custody in Ontario in 2000 compared to general population of Canada in 2006, by sex and primary cancer site. *Standardized for age. †There were no deaths due to colorectal cancer in women in the cohort, and the SMR of 0 is not shown.

The overall cancer-specific mortality rate was 1.0 per 1,000 person-years for men and 0.8 per 1,000 person-years for women (Table 3). The SMR was 1.6 (95% CI 1.4–1.7) in men and 1.4 (95% CI 1.0–1.9) in women (Fig 2). The SMR was higher in men for any cancer, lung cancer, liver cancer, and head and neck cancer, and in women for lung, liver, and head and neck cancers, and the SMR was not lower for any type of cancer in men or women.

## Discussion

This study of men and women who experienced incarceration in Ontario reveals a 10-year cancer prevalence of 0.4% in men and 0.6% in women at the time of admission in 2000. Compared to the general population, the SIR for those who experienced incarceration was higher for lung, liver and head and neck cancers in men, higher for lung, cervical, and liver cancers in women, lower for prostate and colorectal cancers in men, and lower for breast cancer in women. The SMR was increased in men for any cancer, lung cancer, liver cancer, and head and neck cancer, and in women for lung, liver and head and neck cancers.

When comparing our prevalence findings with other studies, we note that the cancer prevalence in incarcerated persons in this study is lower than in previous studies [18–21]. This could reflect that we included only cancer diagnosed in the past 10 years instead of cancer ever [18, 19], the use of self-report [42] in other studies [18–21], differences in the distribution of age or other cancer risk factors leading to a true difference in prevalence, or differences in health care utilization or cancer diagnosis reporting leading to an apparent prevalence difference.

Regarding mortality, the point estimates for the SMRs for any cancer in men and women were similar to those from most other studies [35–37], with the exception of the Georgia study in which the SMR due to any cancer was 1.0 [34]. This difference may be due to the exclusion in that study of those who died within eight years of the index incarceration [34]. Risk factors for death such as injection drug use, hepatitis C infection, and HIV infection are also on the causal pathway for some cancer types, and the high risk of death due to other causes such as homicide, HIV, accidental poisoning and transportation injuries would compete with cancer as a cause of death [37].

The SIRs for prostate and colorectal cancer in men and breast cancer in women with a history of incarceration were significantly decreased. Other than age, which was adjusted for in the calculation of these SIRs, many risk factors for these cancers are more common in people who experience incarceration, including smoking for colorectal cancer [49, 50], alcohol use for colorectal cancer and breast cancer [49–51] and Black race for prostate cancer [52]. Given this, we hypothesize that the relatively decreased incidence may be due in part to underdiagnosis. Further research to elucidate this issue would be valuable, including to examine participation in screening programs and access to health care for diagnosis.

People who experience incarceration bear a disproportionate burden of disease for cancer compared to the general population, in particular for lung, liver, head and neck and cervical cancers. As noted already, many carcinogens associated with these cancers [53] are overrepresented in this population, including tobacco use, alcohol use, HPV, HBV, HCV, and HIV [5, 6]. As the increased relative risk for these cancers is largely consistent across age strata, the high SIR does not reflect an age or cohort effect [47], and therefore, prevention efforts should include all affected age groups. The period of incarceration offers a unique opportunity for prevention, which may be feasible since most cohort members spent a total of more than three months in provincial custody over the follow up period, and limited evidence suggests that prevention initiatives are desired by people in custody [54–56]. Strategies for cancer prevention that could be offered in prisons include HPV and HBV vaccination, smoking cessation treatment, pap screening, linkage with organized screening programs and HCV treatment, and the published literature reveals examples of evaluations of some of these efforts in specific jurisdictions [57–59].

Though prevalent cancer affects only 0.4% of men and 0.6% of women admitted to custody, the majority of these persons have been diagnosed with cancer within the past five years, during which time people with cancer are likely receiving treatment, recovering from treatment, and obtaining close follow up for recurrence and supportive care [45]. Correctional health care must be appropriately structured and resourced to manage and support these patients with significant illness, in collaboration with community partners.

This study has several strengths. The cohort is large and representative. We accessed data on all persons admitted to a provincial correctional facility in 2000, which includes those who were subsequently transferred to the federal system, *i*.*e*. those sentenced to two years or longer. We achieved a high rate of linkage with health administrative data [38]: 97.4%. We expect to have high case ascertainment since the Ontario Cancer Registry collects data on incident cancer cases in Ontario residents from multiple sources [60], and has been shown to have high completeness and validity [61].

There are potential limitations with respect to outcome ascertainment and the sample size. If people who experience incarceration access care less often than the general population and therefore are less likely to have existing cancer diagnosed, our SIRs would be biased toward the null or below one. We do not know whether people who experienced incarceration were more likely to move outside Ontario within the follow up period compared to the general population; if they were, the SIRs and also SMRs would similarly be biased toward the null or below one. Some of the analyses of outcomes in women were likely underpowered, for example the calculation of SMRs for specific cancer sites. This could be associated with Type II error if there were a true difference in mortality between women in the cohort and in the general population, therefore these results should be considered exploratory and interpreted with caution.

## Conclusions

People who experience incarceration are at an increased risk of developing and dying from certain types of cancer. Incarceration presents an opportunity for primary, secondary and tertiary prevention of cancer. We recommend further research to define access to prevention initiatives for this population while in custody and post-release, and knowledge translation efforts to ensure equitable access to and appropriate implementation and evaluation of such programs.

## Supporting information

S1 Appendix Relative risk of incident cancer 2000 to 2012 in persons admitted to provincial correctional facilities in Ontario in 2000 compared to the general population in Ontario in 2006, by age stratum, gender, and cancer type. (DOCX)
