# Supplementary material for: Cancer prevalence, incidence and mortality in people who experience incarceration in Ontario, Canada: A population-based retrospective cohort study
Source: PLoS One. 2017 Feb 22;12(2):e0171131. doi: 10.1371/journal.pone.0171131 (PMC5321272; doi:10.1371/journal.pone.0171131)
Supplement: Supplementary file 1 [file pone.0171131.s001.docx]

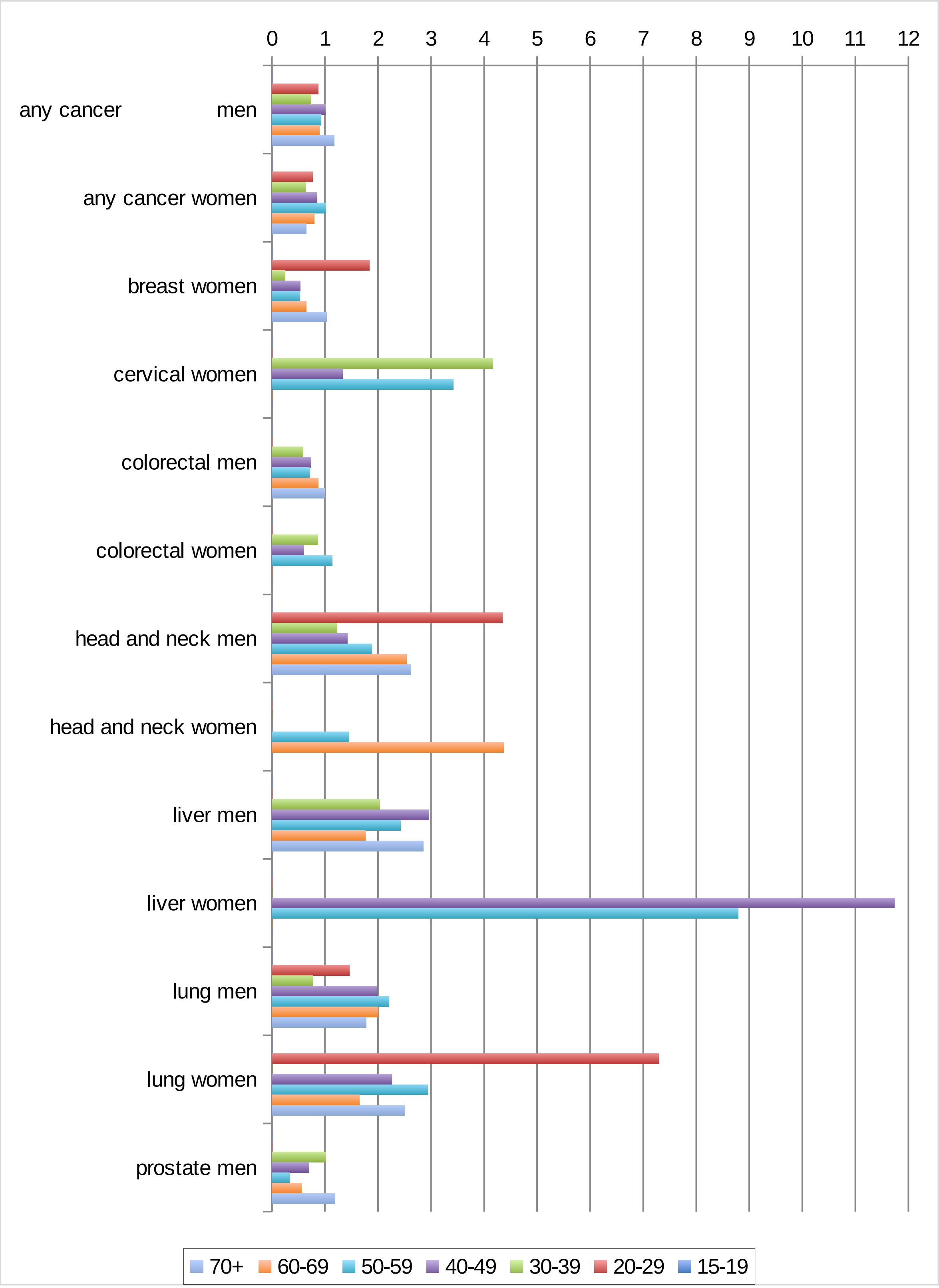

0
1
2
3
4
5
6
7
8
9
10
11
12
any cancer men
any cancer women
breast women
cervical women
colorectal men
colorectal women
head and neck men
head and neck women
liver men
liver women
lung men
lung women
prostate men
70+
60-69
50-59
40-49
30-39
20-29
15-19
